# DEPRESSION, ANXIETY, STRESS, AND HEADACHE IN BRAZILIAN OLDER PEOPLE IN THE CONTEXT OF THE COVID-19 INFODEMIC

**DOI:** 10.1093/geroni/igac059.1795

**Published:** 2022-12-20

**Authors:** Alexandre Bulgarelli, Camila Mello Dos Santos, Ricardo Cavalcante, Karla Frichembruder

**Affiliations:** University of Rio Grande do Sul, PORTO ALEGRE, Rio Grande do Sul, Brazil; University of Rio Grande do Sul, PORTO ALEGRE, Rio Grande do Sul, Brazil; Federal University of Juiz de Fora, PORTO ALEGRE, Rio Grande do Sul, Brazil; University of Rio Grande do Sul, PORTO ALEGRE, Rio Grande do Sul, Brazil

## Abstract

**Objective:**

The aim of the present study was to analyze the prevalence and factors associated with headaches in Brazilian older people in the context of COVID-19 Infodemic.

**Methods:**

This is a cross-sectional study carried out with 3,307 older Brazilians through a virtual questionnaire, self-completed using a cell phone, tablet, or computer with internet access. The questionnaire was composed of the Geriatric Depression Scale(GDS), Geriatric Anxiety Inventory(GAI), and the Perceived Stress Scale (PSS). Data collection was developed between June 2020 and January 2021. The analysis model consisted of variables distributed into four blocks: exogenous variables, primary determinants, health behaviors, and health conditions. It was used the Goodness-of-fit test to assess the quality of fit of the final model. Poisson regression with robust variance was used to estimate the associations.

**Results:**

The prevalence of headache was 31.7% (CI 95%: 30 – 33). This outcome was associated with the use of psychotropic drugs (p < 0,001), concern with information about COVID-19(p < 0,001), symptoms of depression and anxiety(p < 0,001), and perception of stress(p < 0,001).

**Conclusion:**

Anxiety, depression, and stress are thought to be associated with headaches in older adults who are exposed to excess information and fake news about COVID-19. It is considered that in the COVID-19 Infodemic scenario, headache in older people who have access to information is an important marker of mental health associated with suggestions of depression, anxiety, and stress.

